# Metagenome-assembled bacterial genomes recovered from the datasets of *Spodoptera frugiperda* (Smith) (Lepidoptera: Noctuidae)

**DOI:** 10.1016/j.dib.2023.109989

**Published:** 2023-12-18

**Authors:** Francisco Javier Flores Gallardo, José Luis Hernández Flores, Selene Aguilera Aguirre, Miguel Ángel Ramos López, Jackeline Lizzeta Arvizu Gómez, Carlos Saldaña Gutierrez, María Carlota García Gutiérrez, José Alberto Rodríguez Morales, Juan Campos Guillén

**Affiliations:** a Facultad de Química, Universidad Autónoma de Querétaro, Querétaro, Qro 76010, México; b Centro de Investigación y de Estudios Avanzados del IPN, Dpto. de Ingeniería Genética, Irapuato, Gto 36824, México; c Departamento de Química y Bioquímica, Instituto Tecnológico de Tepic/Laboratorio de Microbiología, Laboratorio Integral de Investigación en Alimentos, Tepic, Nayarit 63175, México; d Secretaría de Investigación y Posgrado, Centro Nayarita de Innovación y Transferencia de Tecnología (CENITT), Universidad Autónoma de Nayarit, Tepic, Nay 63173, México; e Facultad de Ciencias Naturales, Universidad Autónoma de Querétaro, Querétaro, Qro 76220, México; f Facultad de Ingeniería, Universidad Autónoma de Querétaro, Querétaro 76010, México

**Keywords:** Armyworm, *Enterococcus casseliflavus*, *Enterococcus mundtii*, *Lactiplantibacillus plantarum*, Metagenome-assembled genomes

## Abstract

*Spodoptera frugiperda* (Smith) (Lepidoptera: Noctuidae), also known as the fall armyworm, is an economically important and widespread polyphagous pest. Microorganisms associated to this insect during life cycle play important ecological roles. We report 3 metagenome-assembled bacterial genomes reconstructed from a metagenome dataset obtained from *S. frugiperda* larvae F3 3rd-instar reared using artificial diet under laboratory conditions. Genome data for *Enterococcus casseliflavus* indicated a genome length of 3,659,8333 bp and GC content of 42.54%. Genome data for *E. mundtii* indicated a genome length of 2,921,701 bp and GC content of 38.37%. Finally, genome data for *Lactiplantibacillus plantarum* indicated a genome length of 3,298,601 bp, GC content of 44.31%. Genome analysis allowed us to identify genus-specific protein families (PLFams), transporters and antibiotic resistance-related genes among others. DNA sequences were deposited in National Center for Biotechnology Information (https://www.ncbi.nlm.nih.gov/) as Bioproject accession PRJNA899064.

Specifications Table

**Table:** 

Subject	Biology
Specific subject area	Microbiology, genomics
Type of data	Tables, Figures
How the data were acquired	The Shotgun DNA sequencing was performed on Illumina NovaSeq Sequencer at Zymo Research, Irvine, CA. Generating 1.1 Gb of paired-end reads of 150 bp in length
Data format	Raw, Analyzed, filtered, and assembled genome sequence
Description of data collection	DNA was extracted from *S. frugiperda* larvae for metagenome sequencing. Genome sequences of 3 bacterial species were reconstructed from the metagenome datasets
Data source location	Institution: Universidad Autónoma de Querétaro City/Town/Region: Querétaro Country: México Latitude and longitude: 20°35′28″N 100°24′36″E
Data accessibility	Repository name: Data was submitted to NCBI GenBank in the public repository. Data identification number: PRJNA899064 Direct URL to data: https://www.ncbi.nlm.nih.gov/bioproject/?term=PRJNA899064 BioSamples: SAMN36465420; SAMN36465421; SAMN36465422

## 1 Value of the Data

• The Genomes of *E. casseliflavus, E. mundtii* and *L. plantarum* can provide insights for the understanding of bacterial interaction with *S. frugiperda.*
• These bacterial genomes data are applicable for comparative genomic and taxonomic purposes.
• These data are valuable resources for researchers working in the field of *S. frugiperda* microbiome to understand ecological interactions and use of biological control agents.
• Data will help to expand the knowledge of bacteria associated to healthy larvae under laboratory-rearing conditions or their interactions with the artificial diet.

## 2 Objective

Healthy colonies of insects are a mandatory requirement for biocontrol experiments. In this regard, endogenous microbiota of *S. frugiperda* might influence growth development and overall state of the insect. However, little is known about *S. frugiperda* microbiota during rearing using artificial diet under laboratory conditions. Therefore, the aim of the present work was to identify relevant genomic features and functional genes from 3rd-instar larvae of *S. frugiperda*-related bacteria with a potential ecological role, through a metagenome-assembled bacterial genome approach.

## 3 Data Description

**Fig. 1:**
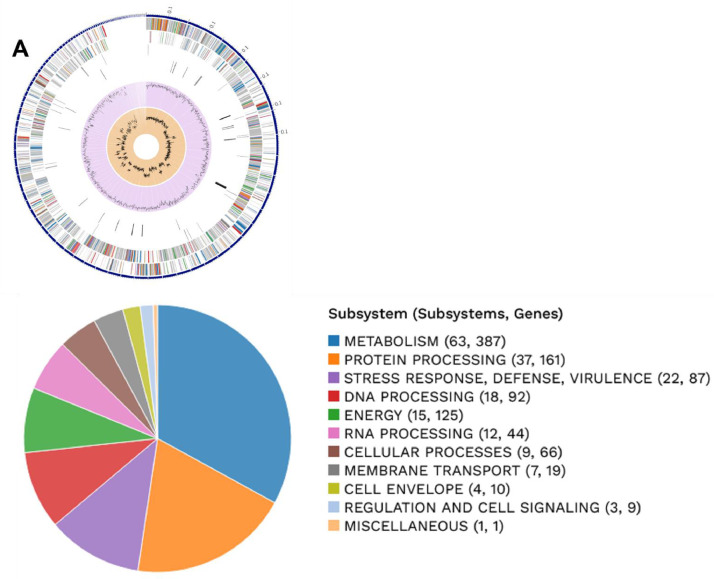

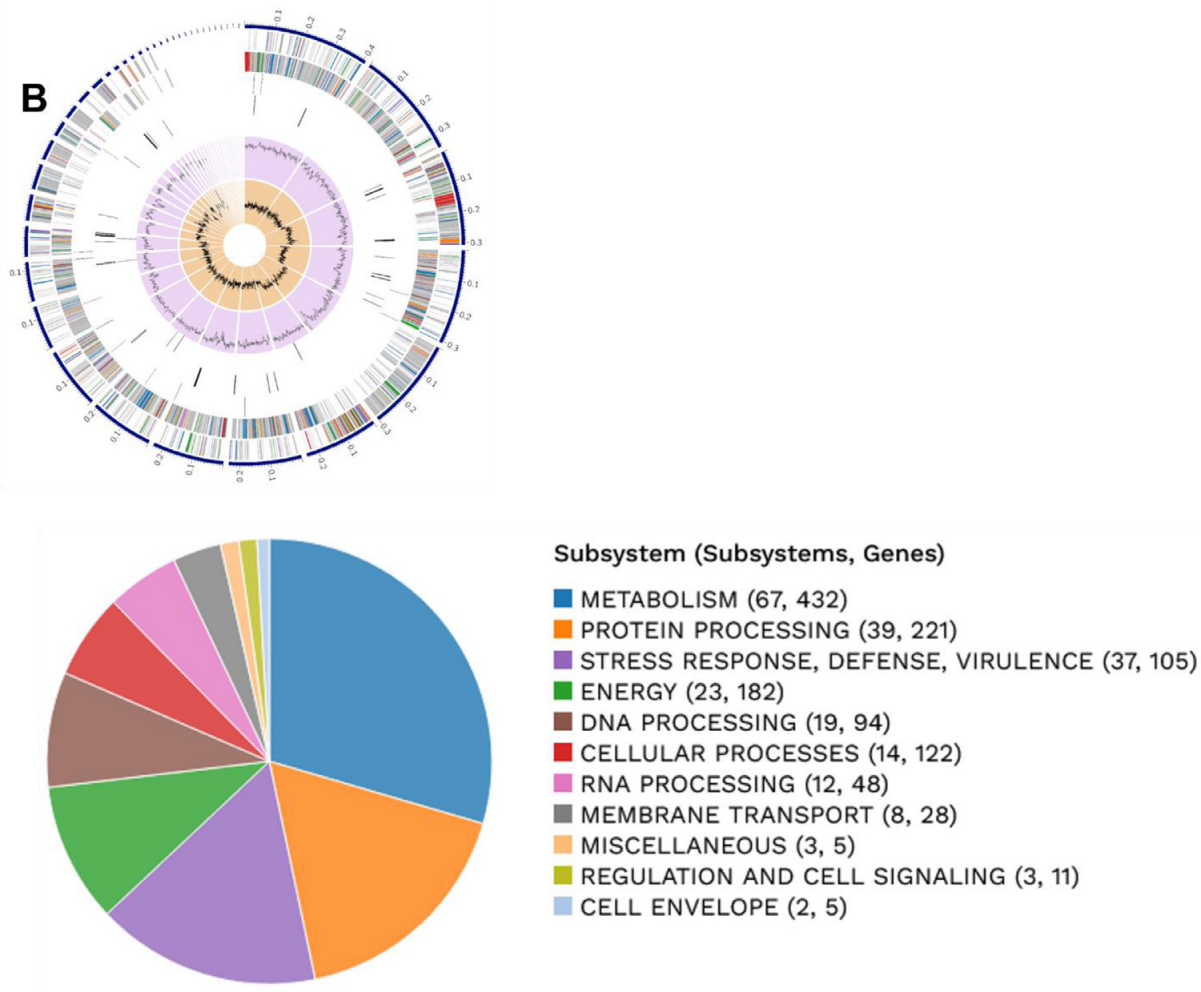

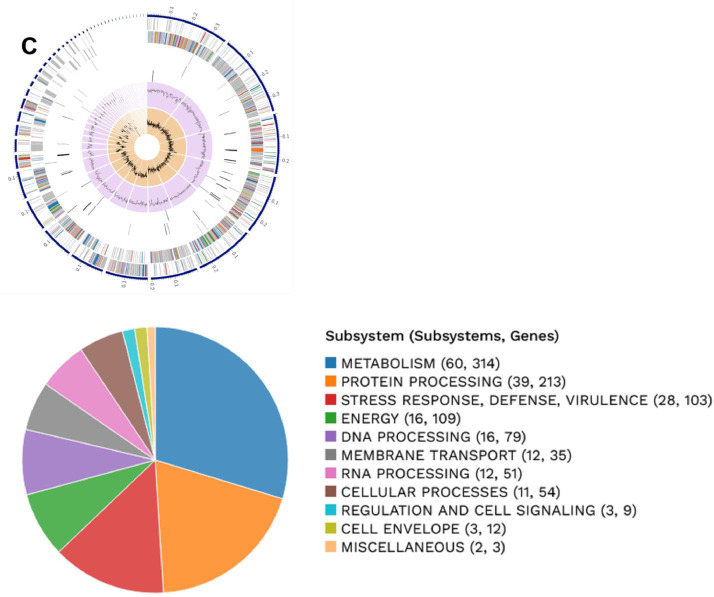
Circular genome map of *Lactiplantibacillus plantarum* (A), *Enterococcus casseliflavus* (B) and *Enterococcus mundtti* (C). The upper figure shows from outer to inner rings, the contigs, CDS on the forward strand, CDS on the reverse strand, antibiotic resistance genes, transporters, GC content and GC skew. The color of the CDS indicates the subsystem that these genes belong. The lower figure is a PATRIC annotation using RAST tool kit (RASTtk), which shows the CDS for subsystem functional assignments to which these genes belong. The numbers provided in parentheses on the right side of the subsystem name indicate the count of subsystems and the count of genes associated with the subsystem name.

This data contains metagenome-assembled bacterial genome using shotgun metagenomic sequencing of two 3rd-instar larvae of *S. frugiperda* reared using artificial diet under laboratory conditions [1,2]. The sequencing result was of 1.1 Gb paired-end reads of 150 bp in length. Table 1 provides the MAGs available in the dataset. Bacterial binning analyzed in CheckM [3] with high-quality produced with > 99.8 % completeness and < 2.1% contamination (Table 1). The BV-BRC metagenomic binning service [3] show that the genome (Fig. 1) for *E. casseliflavus* [4] contains 41 contigs with genome length of 3,659,833 bp, a mean coverage of 272.59 and GC content of 42.54%. The annotated genome identifies 3338 proteins belong to genus-specific protein families (PLFams) and 3664 protein coding sequence (CDS), 1 virulence factor according to VFDB source, 33 transporters and 41 antibiotic resistance-related genes. The genome for *E. mundtii* [5] contains 47 contigs with genome length of 2,921,701 bp, a mean coverage of 378.03 and GC content of 38.37%. The annotated genome identifies 2739 proteins belong to genus-specific protein families (PLFams) and 2923 protein coding sequence (CDS), 2 virulence factor according to VFDB source, 15 transporters and 38 antibiotic resistance-related genes. The genome for *L. plantarum* [6] contains 122 contigs with genome length of 3,298,601 bp, a mean coverage of 14.81 and GC content of 44.31%. The annotated genome identifies 2893 proteins belong to genus-specific protein families (PLFams) and 3258 protein coding sequence (CDS), not detected virulence factor, 16 transporters and 28 antibiotic resistance-related genes. Table 2 lists the antibiotic resistance genes present in each bacteria specie.

**Table 1:** General features of metagenome-assembled bacterial genomes generated from 3rd-instar larvae of *S. frugiperda* reared using artificial diet under laboratory conditions.

Features	*Enterococcus casseliflavus*	*Enterococcus mundtii*	*Lactiplantibacillus plantarum*
MAG identification	bin.2.37734	bin.4.53346	bin.3.1590
Biosample ID	SAMN36465420	SAMN36465422	SAMN36465421
Accession no.	JAUQTC000000000	JAUQTE000000000	JAUQTD000000000
Completeness (%)	100	99.8	100
Contamination (%)	0.8	1.7	2.1
Contig count	41	47	122
DNA size (bp)	3,659,833	2,921,701	3,298,601
Mean Coverage	272.59	378.03	14.81
GC Content (%)	42.54	38.37	44.31
CDS	3664	2923	3258
Proteins with PATRIC genus-specific family (PLfam) assignments	3388	2739	2893
Virulence Factor (VFDB)	1	2	Not detected
Transporter (TCDB)	33	15	16
Antibiotic Resistance genes (PATRIC)	41	38	28

**Table 2:** Antibiotic resistance genes present in each bacterial species annotated from PATRIC database using the pipelines at Bacterial and Viral Bioinformatics Resource Center (BV-BRC).

Antibiotic resistance genes	*Enterococcus casseliflavus*	*Enterococcus mundtii*	*Lactobacillus plantarum*
*aac(6′)-Ie-aph(2”) (and related aacs)*	-	+	-
*alr*	+	+	+
*ddl*	+	+	+
*dfrE*	+	+	-
*fusA*	+	+	+
*efrB*	+	+	-
*tuf*	+	+	+
*fabk*	-	-	+
*folA*	+	+	+
*folp*	-	-	+
*fosX* *gdpD* *gidB* *gyrA* *gyrB* *inhA, fabL* *ileS* *kasA* *liaF* *liaR* *liaS* *mprF* *murA* *pgsA* *rho* *rlmA(II)* *rpoB* *rpoC* *rpsJ* *rpsL* *OmpR* *VanR* *VanC* *VanR* *BaeS* *vanT* *vanXY*	+ + + + + + + + + + + + + + + + + + + + + + + + + + +	- + + + + + + + + + + + + + + + + + + + + - - - - - +	- + + + + + + + - - - + + + + + + + + + + - - - - - -

## 4 Experimental Design, Materials and Methods

### 4.1 Rearing of *S. frugiperda*

In this study, *S. frugiperda* (instar L4-L5) were freshly collected from maize field crops during 2022, transported in containers, and reared using artificial diet under laboratory conditions [1,2].

### 4.2 DNA Extraction

For DNA extraction and metagenomics analysis, two complete F3 larvae (instar L3) were selected to obtain only one DNA sample. DNA was isolated using the ZymoBIOMICS DNA Miniprep Kit (Zymo Research, Irvine, CA) following the manufacturer's instructions. The genomic DNA was processed and analyzed with the Shotgun Metagenomic Sequencing Service (Zymo Research, Irvine, CA).

### 4.3 Sequencing and Assembly

Sequencing libraries were prepared with Illumina® DNA Library Prep Kit (Illumina, San Diego, CA) and the final library was sequenced on the platform NovaSeq® (Illumina, San Diego, CA). Generating 1.1 Gb of paired-end reads of 150 bp in length. Bioinformatics analyses were made using the pipelines at Bacterial and Viral Bioinformatics Resource Center (BV-BRC) and was submitted to the Metagenomic Binning Service [3,[7], [8], [9], [10]. Each set of binned contigs was annotated using RAST tool kit (RASTtk) [10]. All software were run with default parameters.

## Data Accessibility

The raw sequence data were deposited at the National Centre for Biotechnology Information (NCBI) database under the project number PRJNA899064. The sequences of MAGs are available at GenBank under the genome accessions summarized in Table 1.

## Ethics Statements

This work did not involve any human subjects, animals or species that require ethical approval.

## CRediT authorship contribution statement

**Francisco Javier Flores Gallardo:** Methodology, Software. **José Luis Hernández Flores:** Conceptualization, Methodology, Software, Writing – original draft. **Selene Aguilera Aguirre:** Methodology, Software, Formal analysis. **Miguel Ángel Ramos López:** Writing – original draft. **Jackeline Lizzeta Arvizu Gómez:** Investigation, Formal analysis. **Carlos Saldaña Gutierrez:** Investigation. **María Carlota García Gutiérrez:** Methodology. **José Alberto Rodríguez Morales:** Methodology. **Juan Campos Guillén:** Conceptualization, Methodology, Software, Writing – original draft.

## Data Availability

PRJNA899064 (Original data) (NCBI GenBank).
